# Control and Prevention of Anthrax, Texas, USA, 2019

**DOI:** 10.3201/eid2612.200470

**Published:** 2020-12

**Authors:** Tom Sidwa, Johanna S. Salzer, Rita Traxler, Erin Swaney, Marcus L. Sims, Pam Bradshaw, Briana J. O’Sullivan, Kathy Parker, Kenneth A. Waldrup, William A. Bower, Kate Hendricks

**Affiliations:** Texas Department of State Health Services, Austin, Texas, USA (T. Sidwa, E. Swaney, B.J. O’Sullivan, K. Parker);; Centers for Disease Control and Prevention, Atlanta, Georgia, USA (J.S. Salzer, R. Traxler, W.A. Bower, K. Hendricks);; Shannon Health System, Ozona, Texas, USA (M.L. Sims);; Shannon Medical Center, San Angelo, Texas, USA (P. Bradshaw);; Texas Department of State Health Services, El Paso, Texas, USA (K.A. Waldrup)

**Keywords:** anthrax, treatment, prophylaxis, prevention, vaccination, infection control, zoonoses, vaccine-preventable diseases, Texas, bacteria, Bacillus anthracis

## Abstract

The zoonotic disease anthrax is endemic to most continents. It is a disease of herbivores that incidentally infects humans through contact with animals that are ill or have died from anthrax or through contact with *Bacillus anthracis*–contaminated byproducts. In the United States, human risk is primarily associated with handling carcasses of hoofstock that have died of anthrax; the primary risk for herbivores is ingestion of *B. anthracis* spores, which can persist in suitable alkaline soils in a corridor from Texas through Montana. The last known naturally occurring human case of cutaneous anthrax associated with livestock exposure in the United States was reported from South Dakota in 2002. Texas experienced an increase of animal cases in 2019 and consequently higher than usual human risk. We describe the animal outbreak that occurred in southwest Texas beginning in June 2019 and an associated human case. Primary prevention in humans is achieved through control of animal anthrax.

The zoonotic disease anthrax, caused by the bacterium *Bacillus anthracis*, has been known to humankind for thousands of years and is endemic to most continents (*1*–*3*). It is a naturally occurring disease of herbivores that incidentally infects humans through contact with animals that are ill or have died from anthrax or through contact with *B. anthracis*–contaminated byproducts such as meat, hides, hair, and wool (*4*). Transmission routes include cutaneous, ingestion, inhalation, and injection; cutaneous accounts for most (95%) cases worldwide (*2*,*4*). In the United States, human risk is primarily associated with handling carcasses of hoofstock that have died of anthrax; the primary risk for herbivores is ingestion of *B. anthracis* spores that can persist in suitable alkaline soils in a corridor from Texas through Colorado, the Dakotas, and Montana (*5*–*7*).

The 2 state agencies responsible for anthrax surveillance in Texas are the Texas Department of State Health Services (DSHS) and the Texas Animal Health Commission (TAHC). Samples that are culture-positive for *B. anthracis* at veterinary reference laboratories are reported to DSHS and TAHC. Veterinarians treating animals with illnesses compatible with anthrax must also report to DSHS and TAHC. Suspected cases of human anthrax are immediately reportable to DSHS. Samples or isolates from human cases are forwarded for identification to local public health reference laboratories. In Texas, animal anthrax cases are most commonly reported from the triangular area bounded by the towns of Uvalde, Ozona, and Eagle Pass (Figure 1), which includes portions of Crockett, Val Verde, Sutton, Edwards, Kinney, Uvalde, Zavala, and Maverick Counties in southwestern Texas.

**Figure 1:**
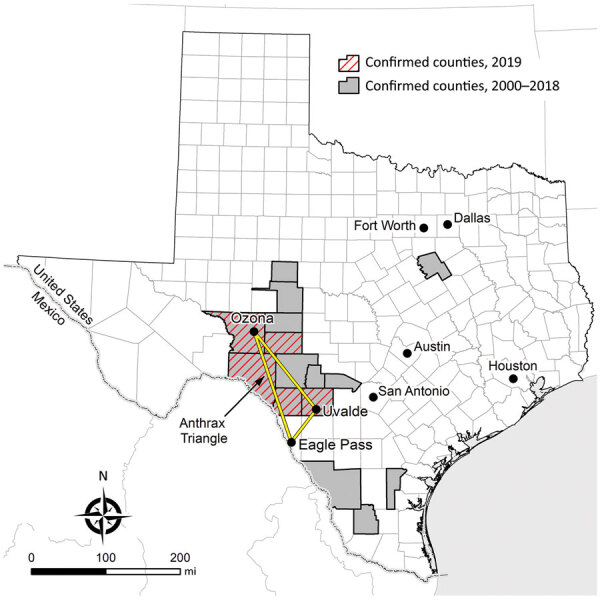
Counties with confirmed animal anthrax cases, Texas, USA, 2000–2019. The location of the “Anthrax Triangle” is indicated.

During 2000–2018, a total of 63 animal anthrax cases were confirmed by culture of *B. anthracis* in a reference laboratory (annual mean 3.3, range 0–20 cases/year) (T. Sidwa, unpub. data). Because only 1 animal per affected premise usually is reported in a given year, the number of cases is a substantial underrepresention of the total number of affected animals and properties. The last naturally occurring human case of cutaneous anthrax associated with livestock exposure in Texas was reported in 2001 (*8*,*9*).

## Texas Outbreak 2019

### Animal Cases

Texas Veterinary Medical Diagnostic Laboratory confirmed the first anthrax case of 2019 in an exotic antelope carcass from Uvalde County on June 19. Overall in 2019, the laboratory reported 25 culture-positive animals, including cattle, horses, white-tailed deer, antelope, and a goat, from Crockett, Kinney, Sutton, Uvalde, and Val Verde counties. The last confirmed animal case was reported on August 21. Unconfirmed numbers reported to DSHS staff suggest that >1,000 animal losses might be attributed to the 2019 outbreak (K. Waldrup, unpub. data).

Implementing control measures (i.e., vaccination and proper carcass disposal) was challenging; thin topsoil over bedrock, vast and inaccessible terrain, and burn bans triggered by hot, dry weather conditions made it difficult for livestock owners and landowners to identify and bury or burn dead animals. Livestock owners can sometimes cover dead animals with tarps if burial or burning is not an option. However, because properties in this area of Texas can be thousands of acres and not particularly navigable, reaching dead animals to cover and protect them from scavengers (that might further distribute *B. anthracis*–contaminated remains) is often not feasible.

Another obstacle to controlling the outbreak was the inability to address the contribution of wildlife to the initiation and perpetuation of disease spread (e.g., lack of a licensed vaccine and impracticality of using physical or chemical restraint to administer vaccine “off label” to wildlife species). In addition, reports of vaccine-associated adverse events among goats and horses (*2*,*10*) made some owners reluctant to vaccinate these species. Among confirmed animal anthrax cases in species for which vaccination is indicated (cattle, goats, horses, sheep, and swine) (*11*), a third are reported to have been vaccinated before illness. Of those, the median number of days from most recent vaccination to specimen collection was 8 days (range 3–82 days) (T. Sidwa, unpub. data). The frequency and effect of antibiotic use subsequent or simultaneous to vaccination was unknown.

### Human Case Report

On July 23, 2019, a non-Hispanic White man in his 70s from the anthrax-affected area who had a history of cardiovascular disease and hypertension visited his physician for evaluation of 2 lesions near his right knee. Four days earlier, a small red spot had emerged and gradually enlarged and became painful. He reported no fever and used no over-the-counter medications. When asked about animal exposures because of where he lived, he reported that he and his daughter had moved 2 fly-covered deer carcasses from beneath his porch before lesion onset. He was wearing shorts and a shirt while moving the carcasses, and his affected leg was scraped by the velvet-covered antlers. He also reported being bitten by a fly. The deer carcasses were not tested for anthrax, and the patient disposed of them.

**Figure 2:**
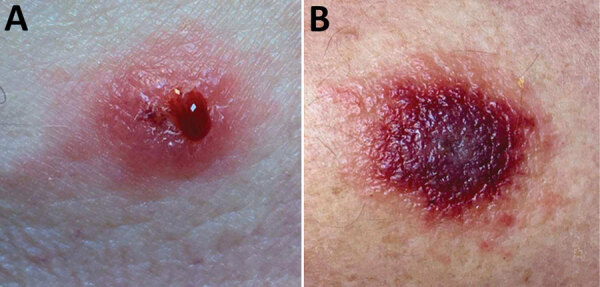
Lesions on right leg of anthrax patient as seen on outpatient visit, Texas, USA, 2019.

On examination at his physician’s office, the patient’s vital signs were as follows: blood pressure 177/87 mm Hg; heart rate 76 beats/min; and temperature 98.3°F. Below and lateral to his right knee was an indurated, raised, erythematous 5-cm lesion with small ulcerations that oozed serosanguinous fluid and was surrounded by a blanched halo. Just proximal to his right knee was a nonindurated erythematous macule (Figure 2). No popliteal or inguinal adenopathy was present. After 2 swab specimens were obtained from the larger lesion, the patient was given a cephalosporin intramuscularly, and a prescription for ciprofloxacin was called in to his pharmacy of choice more than an hour’s drive from his home. Because it was too late to send the specimens anywhere for testing on that day, the swabs were mailed directly to the Texas Department of State Health Services Laboratory on Wednesday after a phone consultation with the state health department.

**Figure 3:**
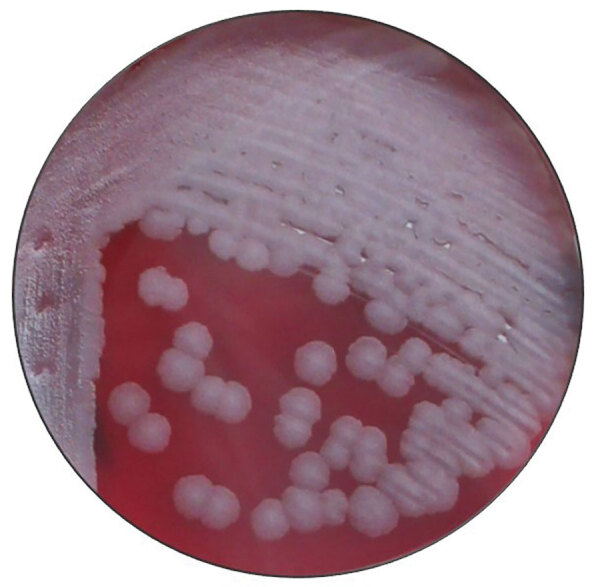
*Bacillus anthracis* 24-hour growth on sheep blood agar from a swab of a cutaneous anthrax lesion from a patient in Texas, USA, 2019. Typical ground glass colony morphology and lack of hemolysis are shown.

**Figure 4:**
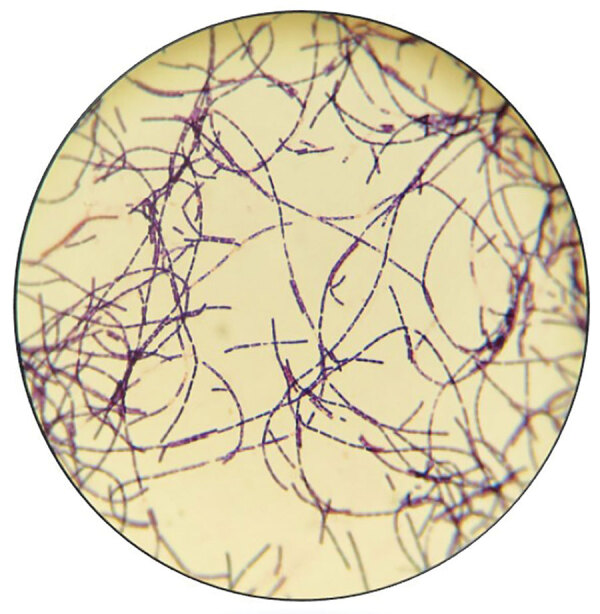
Gram stain from culture of a lesion of an anthrax patient, Texas, USA, 2019.

The patient began his ciprofloxacin the next evening (July 24). On July 26, after having taken 4 doses of his antibiotics, he was feeling worse and sought additional care at the emergency department of hospital A, more than an hour’s drive from his residence. Concurrently, the state laboratory notified his primary-care physician that a preliminary laboratory report for the specimen was PCR-positive for *B. anthracis*; this result was confirmed by culture the following week (August 1) (Figures 3, 4). His physician relayed the information first to the patient and then to hospital staff. Upon arrival to the hospital, the patient reported pain, difficulty walking, and nausea. He reported intermittent spontaneous drainage of a dark, jelly-like material from the larger wound. He reported no fever, chills, chest pain, shortness of breath, pain at rest, numbness, or tingling. He did not use tobacco products.

**Figure 5:**
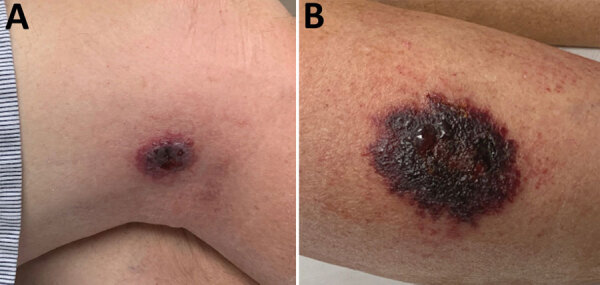
Eschars on right leg of anthrax patient as seen at hospital admission, Texas, USA, 2019.

At hospital A, he reported that his exposure had been »3 weeks earlier. At examination, his vital signs were blood pressure 132/71 mm Hg; heart rate 91 beats/min; and respirations 24 breaths/min. He was afebrile. He had a nondraining, nonerythematous eschar 7.2 cm × 5 cm on the lateral aspect of the right calf and a painless, nondraining, nonerythematous 3.3 cm × 2 cm eschar on the lateral aspect of the right knee (Figure 5). His leukocyte count was 12,000 (10^3^ cells/µL); hemoglobin, 15.5 g/dL; hematocrit, 46.9% g/dL; platelets, 83,000 (10^3^ cells/mL); blood urea nitrogen, 35 mg/dL; and creatinine, 2.6 mg/dL. His antibiotic was switched to intravenous doxycycline (100 mg every 12 hours). He was discharged on hospital day 13.

## Control and Prevention Measures

### Control Measures for Animal Outbreaks

Because naturally occurring human anthrax cases in endemic countries are almost always related to exposure to infected animals or their byproducts, control of animal anthrax essentially eliminates human risk. The primary control measure for animal anthrax is annual preventive vaccination; however, once an outbreak occurs, other control measures include ring vaccination, proper carcass disposal to avoid further environmental contamination, and quarantine (i.e., limit animal movement from the affected and nearby properties, animal contact with anthrax-contaminated sites, and contact between affected and nonaffected herds) (*9*). On the basis of anecdotal reports and 1 small study, tabanid flies (e.g., deer and horse flies) might play a role in transmission; whether fly control is achievable or would be effective remains an open question (*2*,*12*,*13*).

The attenuated Sterne-strain of *B. anthracis* is used globally for vaccination among domestic livestock (*14*). Because the vaccine is live-attenuated, concurrent antibiotic administration can substantially diminish efficacy. If an animal is given antibiotics either 10 days before or after vaccination, revaccination is recommended (*9*,*15*). Whether concurrent administration of antibiotics played a role in diminished vaccine efficacy in the Texas outbreak is unclear.

Proper and safe carcass disposal is critical for controlling anthrax outbreaks in enzootic areas because inappropriate carcass disposal seeds the soil with spores and increases the risk for future epizootics. Global recommendations (*9*) and codified Texas regulations (*16*) for carcass disposal are similar: the carcass should be burned in place, using a pyre or other method that leaves only ash and allows the destruction of the contaminated soil as well (i.e., “burnt until it is thoroughly consumed”) (*9*,*16*). When a carcass cannot be burned, global recommendations are to bury it deeply (*9*). The historic practice of adding lime should be avoided (*17*). High soil calcium levels, either from the addition of lime or as occur naturally in southwest Texas, are conducive to *B. anthracis* spore survival (*6*,*7*) and increase the likelihood of future outbreaks. The least desirable disposal method is leaving the carcass in place, because scavenging can further disseminate the spores and increase future exposure risks for susceptible animals. Alternative carcass disposal methods are needed in areas where the standard recommendations to burn or bury carcasses are impractical. This need is particularly pronounced where there is an abundance of susceptible wildlife species that are not vaccinated or where there is poor vaccination coverage of domestic hoofstock.

### Prevention of Human Cases in Endemic Areas

Human and animal health authorities should remind at-risk populations of the following prevention measures when animal cases are first identified. During animal outbreaks of anthrax, persons who handle and dispose of infected animals are at highest risk for exposure. However, exposure can be minimized through use of personal protective equipment, which should include gloves that can be disinfected or disposed of, long sleeves and pants, and footwear suitable to the terrain that can be disinfected (*9*). Even in the absence of a recognized anthrax outbreak, veterinarians and ranchers in endemic areas should always keep anthrax in mind as they interact with members of susceptible species that are ill. To do otherwise can result in inadvertent exposure to anthrax.

Antibiotic postexposure prophylaxis (PEP) is another important component of prevention. In the former Soviet Union, before 1965, 58/339 (17%) of patients who did not receive antibiotic prophylaxis after cutaneous exposures had onset of anthrax; in contrast, only 5/287 (2%) who received prophylaxis had onset of anthrax (*18*).

If skin or mucus membrane contact occurs during carcass disposal, persons should seek medical attention and receive antibiotic PEP for 7 days (Table 1) and have their symptoms monitored for 14 days. Although aerosol exposure is unlikely in cases of natural cutaneous exposures, if potential aerosol exposure also occurred, antibiotic PEP should be administered for up to 60 days and anthrax vaccine may be considered.

**Table 1:** Oral antimicrobial drugss for postexposure prophylaxis and treatment of localized cutaneous anthrax*

Postexposure prophylaxis alone or after oral or intravenous therapy		Monotherapy for localized cutaneous anthrax
Antimicrobial drugs before susceptibility testing		For all strains, regardless of penicillin susceptibility or if susceptibility is unknown
** Ciprofloxacin 500 mg every 12 h**		** Ciprofloxacin 500 mg every 12 h**
OR		OR
** Doxycycline 100 mg every 12 h**		** Doxycycline 100 mg every 12 h**
OR		OR
Levofloxacin 750 mg every 24 h		Levofloxacin 750 mg every 24 h
OR		OR
Moxifloxacin 400 mg every 24 h		Moxifloxacin 400 mg every 24 h
OR		OR
Clindamycin† 600 mg every 8 h		Clindamycin† 600 mg every 8 h
OR		OR
For penicillin-susceptible strains		For penicillin-susceptible strains
Amoxicillin 1 g every 8 h		Amoxicillin 1 g every 8 h
OR		OR
Penicillin VK 500 mg every 6 h		Penicillin VK 500 mg every 6 h
Because patients who have had aerosol exposures might still have residual spores in their lungs even after treatment, oral postexposure prophylaxis is recommended as follows: for noncases (i.e., no treatment) without AVA, 60 d; with AVA for healthy adults 18–65 y, 14 d after the 3rd dose of AVA; with AVA for children <18 y, adults >65 y, pregnant women, and adults with underlying conditions, 60 d. For cases (i.e., following treatment) after finishing oral or intravenous treatment, patients exposed to aerosolized spores should finish out a 60-d course of antimicrobials (i.e., 60 d minus the duration of treatment)		Duration of therapy for naturally acquired cases, 7 d

*Bold type indicates preferred agent. Nonbolded type indicates alternative selections, which are listed in order of preference for therapy for patients who cannot tolerate first-line therapy or if first-line therapy is unavailable. †Based on in vitro susceptibility data, rather than studies of clinical efficacy.

Persons who live and work in anthrax-endemic areas and who anticipate interacting with animals that are dying or have died of anthrax might wish to consider preexposure prophylaxis with anthrax vaccine adsorbed (AVA). For preexposure prophylaxis of persons at high risk for *B. anthracis* exposure, AVA is administered intramuscularly as a priming series at 0, 1, and 6 months, with booster doses at 12 and 18 months and annually thereafter (*19*). Health departments in endemic areas that have existing vaccination programs can acquire AVA from the manufacturer.

### Healthcare Infection Control Issues for Cutaneous Anthrax

A person with cutaneous or other type of anthrax (e.g., injection, ingestion, or inhalation) cannot transmit disease through aerosol or droplet. However, spores that could remain on a person’s skin, hair, or clothing after an exposure before they bathe or shower and change clothes might possibly transfer to someone else’s skin and cause cutaneous anthrax (*20*–*22*). Although incubation periods of <1 day are reported, patients usually wait a few days to seek care, making it likely that they would already have bathed and changed clothes before seeking care. It is therefore unlikely that healthcare personnel would be secondarily exposed to spores.

Although cutaneous anthrax lesions can be contagious before the institution of effective antibiotic therapy, they become sterile in <1 day once therapy has begun (*23*). Lesions should be covered until the patient has had 24 hours of effective antibiotics. Contact precautions should be used for the first day; after that, standard precautions are sufficient.

Disposable items that have been in direct contact with the anthrax lesion, any tissue removed during debridement, and potentially infectious wound care materials (*24*,*25*) should be disposed of in a biohazard bag according to guidelines for disposal of any potentially infectious material. No additional disinfection is needed beyond what is regularly scheduled for the facility. Nondisposable surfaces in direct contact with the anthrax lesion or wound drainage can be disinfected with a 0.5% hypochlorite solution, a commercial product such as SporGon (Decon Labs, https://deconlabs.com), or other sporicidal agents such as an Environmental Protection Agency–registered antimicrobial product effective against *B. anthracis* spores (*26*–*28*); products effective against *Clostridium difficile* spores might also be appropriate (*29*,*30*)

### Diagnosis

Although an eschar is the cardinal sign of cutaneous anthrax, in its early stages, anthrax can manifest as a group of small vesicles that might be pruritic. The lesion might be surrounded by erythema and swelling but is usually painless. Lymphadenopathy can occur, and constitutional symptoms including fever and headache are also possible. Localized cutaneous anthrax can disseminate to become a systemic disease. Although a substantial portion (10%–40%) of patients with cutaneous anthrax would die if left untreated (*4*), most can recover with treatment (*31*). Meningitis is also a possible, and typically fatal, complication (*32*).

In the United States, cutaneous anthrax is decidedly rare: other causes of eschars and eschar-like lesions include poxvirus infections (e.g., cowpox, vaccinia, orf), rickettsial infections (e.g., scrub typhus and *Rickettsia parkeri* rickettsiosis), ulceroglandular tularemia, staphylococcal or streptococcal infections, and noninfectious causes such as insect or spider bites. Obtaining a good exposure history is key to determining the likelihood of various etiologies among the differential diagnoses and determining the best specimens to collect. Patients seeking care with an eschar or eschar-like lesion should be asked about recent exposure to dead or dying herbivores or biting flies in an anthrax enzootic area; recent animal bites or scratches; and recent contact with lagomorphs, rodents, fleas, ticks, and spiders.

A Gram stain of a swab specimen from the lesion can often quickly identify possible cases and narrow the differential diagnosis (*23*). Specimens for tests such as Gram stain, culture, and PCR to rule anthrax in or out (Table 2) must be collected before the use of antibiotic therapy because they will rapidly become negative after the implementation of therapy (*23*). Specimens can be sent to sentinel laboratories for preliminary assessment. Specimens for which *B. anthracis* is not ruled out by a sentinel laboratory should promptly be sent to a Laboratory Response Network (LRN) laboratory for confirmation (*33*). LRN is a network of laboratories established to respond to biologic and chemical threats and other public health emergencies that consists of 3 types of laboratories. Private and commercial laboratories comprise the first tier of the LRN and are described as sentinel laboratories. Laboratories that receive reagents, protocols, and specialized training to perform confirmatory testing for multiple agents in high-risk environmental or clinical samples comprise the second tier of LRN and are referred to as reference laboratories. Specialized characterization of organisms, bioforensics, select agent activity, and handling of highly infectious biologic agents is performed at national laboratories, the third tier of LRN. However, with approval from public health authorities, specimens from lesions that are highly suspicious based on clinical or epidemiologic grounds can be sent directly from clinicians to an LRN laboratory (*34*).

**Table 2:** Diagnostic specimens for cutaneous anthrax (*33*)*

Specimen	Test	Temperature	Laboratory Response Network level
1 swab†	Gram stain‡ and culture	Room temperature	Sentinel laboratory§
1 swab†	PCR	Room temperature	Reference laboratory¶
Single plasma or serum	Lethal factor	Frozen (−70°)	CDC#
Paired serum**	Antiprotective antigen	Frozen (−70^0^)	CDC
Full thickness punch biopsy of lesion	Immunohistochemistry	Room temperature	CDC

*CDC, Centers for Disease Control and Prevention. †Dry dacron swabs for swabbing moist lesions (e.g., bullae) or saline-moistened dacron swabs for swabbing beneath dry lesions (i.e., eschars) to be collected before onset of antimicrobial therapy. ‡Direct smear from lesion. §Sentinel laboratories comprise the first level of the Laboratory Response Network; they include private and commercial laboratories that provide routine diagnostic services, rule-out, and referral steps in the identification process. ¶Reference laboratories, often called Laboratory Response Network member laboratories, are responsible for investigating, confirming, or referring specimens. These laboratories perform testing for multiple agents in high-risk environmental or clinical samples. #CDC laboratories belong to the top tier of the Laboratory Response Network (national laboratories). **Acute and convalescent collected 2 weeks apart.

### Notification

Clinicians should promptly notify their local or state health department when they suspect anthrax, although the mandated timing varies by jurisdiction. State and territorial health departments should notify the Centers for Disease Control and Prevention (CDC) within 4–24 hours (*24*) of the initial report for patients whose illness meets the probable or confirmed case definition (*35*). Presumptive positive results from an LRN laboratory must be reported within 2 hours to the state and CDC.

### Treatment

Cutaneous anthrax lacking systemic manifestations such as fever, tachycardia, tachypnea, hypotension, leukocytosis, or leukopenia can usually be treated with 7 days of an oral antibiotic. Patients with cutaneous anthrax should only continue oral antibiotics for PEP after antibiotic treatment is complete if the patient was also exposed to aerosolized spores; this would rarely be indicated for naturally acquired cutaneous infections because aerosol exposures are unlikely (Table 1).

Systemically ill patients should be evaluated for meningitis; if meningitis can be ruled out, they should be treated with at least 2 intravenous antibiotics (1 that is bactericidal and 1 that inhibits protein synthesis to block toxin production). Antibiotic therapy should continue for >2 weeks or until the patient is stable. If meningitis is present, >3 antibiotics should be used (>1 should be bactericidal, >1 should inhibit protein synthesis, and all should have good central nervous system penetration). Antibiotic options for treatment and prevention of anthrax are listed in Tables 1 and 3.

**Table 3:** Intravenous antimicrobials for treatment of adults with severe anthrax*

Dual therapy for when meningitis has been excluded		Triple therapy for when meningitis might be present
Bactericidal agent		Bactericidal agent (fluoroquinolone)
Antimicrobial drugs before susceptibility testing		
** Ciprofloxacin 400 mg every 8 h†**		** Ciprofloxacin 400 mg every 8 h†**
OR		OR
Levofloxacin 750 mg every 24 h		Levofloxacin 750 mg every 24 h
OR		OR
Moxifloxacin 400 mg every 24 h		Moxifloxacin 400 mg every 24 h
OR		PLUS
Meropenem 2 g every 8 h		Bactericidal agent (beta-lactam)
OR		For all strains, regardless of penicillin susceptibility or if susceptibility is unknown
Imipenem‡ 1 g every 6 h		
OR		** Meropenem 2 g every 8 h**
Doripenem 500 mg every 8 h		OR
OR		Imipenem‡ 1 g every 6 h
Vancomycin 60 mg/kg/day divided every 8 h (maintain serum trough concentrations of 15–20 µg/mL)		OR
		Doripenem 500 mg every 8 h
OR		OR
For penicillin-susceptible strains		For penicillin-susceptible strains
Penicillin G 4 million units every 4 h		Penicillin G 4 million units every 4 h
OR		OR
Ampicillin 3 g every 6 h		Ampicillin 3 g every 6 h
**PLUS**		**PLUS**
Protein synthesis inhibitor		Protein synthesis inhibitor
** Clindamycin 900 mg every 8 h**		** Linezolid§ 600 mg every 12 h**
OR		OR
** Linezolid§ 600 mg every 12 h**		Clindamycin 900 mg every 8 h
OR		OR
Doxycycline¶ 200 mg initially, then 100 mg every 12 h		Rifampin# 600 mg every 12 h
OR		OR
Rifampin# 600 mg every 12 h		Chloramphenicol** 1 g every 6–8 h
Duration of therapy for 10–14 d or until clinical criteria for stability are met. Patient exposed to aerosolized spores will require prophylaxis to complete an antimicrobial course of up to 60 d from onset of illness (see postexposure prophylaxis in Table 1)		Duration of therapy for 2–3 weeks or greater, until clinical criteria for stability are met. Patients exposed to aerosolized spores will require prophylaxis to complete an antimicrobial course of up to 60 d from onset of illness (see postexposure prophylaxis in Table 1)

*Bold type indicates preferred agent. Nonbolded type indicates alternative selections, which are listed in order of preference for therapy for patients who cannot tolerate first-line therapy or if first-line therapy is unavailable. †Severe anthrax includes anthrax meningitis, inhalation, injection, and gastrointestinal anthrax; and cutaneous anthrax with systemic involvement, extensive edema, or lesions of the head or neck. ‡Increased risk for seizures associated with imipenem/cilastatin therapy. §Linezolid should be used with caution in patients with thrombocytopenia because it might exacerbate it. Linezolid use for >14 d carries additional risk for hematopoietic toxicity. ¶A single 10–14 d course of doxycycline is not routinely associated with tooth-staining. #**Rifampin is not a protein synthesis inhibitor, it may also be used in combination therapy based on in vitro synergy. **Should only be used if other options are not available, due to toxicity concerns.

Systemically ill patients (whether from cutaneous, ingestion, inhalation, or injection exposures) are candidates for 1 of the Food and Drug Administration–approved anthrax antitoxins. The antitoxins are available through the Strategic National Stockpile pending a consultation with an anthrax subject matter expert at CDC, which can be reached by calling the Emergency Operations Center (770-488-7100).

Surgery might occasionally be indicated for lesions complicated by compartment syndrome. However, surgery usually is not necessary for cutaneous anthrax (*36*).

## Public Health Implications and Conclusion

Anthrax is endemic to parts of the United States. Epizootics emerge with varying frequency when climatic conditions favor the uncovering of soilborne *B. anthracis* spores with subsequent consumption by susceptible herbivores. Humans contract cutaneous anthrax through contact with animals that are ill or have died from anthrax or contact with *B. anthracis*–contaminated byproducts; this risk is increased during epizootics. The outbreak we describe was confirmed in June 2019, but its actual start date is unknown; reliable recognition of epizootics might be impeded when they occur in vast, rough, and sparsely populated areas such as those in the anthrax-endemic areas of Texas. These same geographic characteristics create challenges in implementing the recommended disease control interventions, including appropriate carcass disposal and broad use of animal anthrax vaccine in species for which the vaccine is licensed, as well as off-label use in other species. Wild herbivores (e.g., white-tailed deer and exotic hoofstock) contributed to the 2019 Texas outbreak, but effective mitigation (carcass disposal or vaccination) of the risk they posed could not be adequately achieved.

The cutaneous anthrax patient associated with this outbreak was apparently exposed through a scratch on the leg from the antler of an untested deer carcass. The physician he visited in rural Texas included anthrax in the differential diagnosis, obtained and submitted diagnostic samples before treating the patient, and provided the patient with a prescription for oral ciprofloxacin. Anthrax was identified through PCR and confirmed through culture at the state reference laboratory from swab specimens of a leg lesion. The patient was treated as an outpatient with appropriate antibiotics until his condition worsened and required a 13-day hospitalization. The necessity for hospitalization might have been related to a few-week delay in seeking treatment. Despite the delay, the patient, like most patients with cutaneous anthrax, survived with antibiotic treatment (*4*,*32*).

As soon as anthrax is recognized in an animal population, public health and animal health agencies must collaborate to heighten awareness among medical and animal health communities, as well as among ranchers and other inhabitants of at-risk areas. Timely delivery of information to ranchers on proper carcass disposal and appropriate use of personal protective equipment, as was done through various alerts, might reduce the number of exposures. If exposure is recognized, antibiotic PEP should be considered by medical providers. AVA may be appropriate for persons at high risk for exposure, such as veterinary staff and ranch workers in endemic areas; however, this process involves a long-term commitment to annual booster shots to ensure protection.

Ranchers and veterinarians should receive authoritative information on animal vaccine use to break the cycle of transmission (including emphasis on avoiding administration of antibiotics 10 days before or after vaccine administration). Even in the absence of a recognized anthrax outbreak, veterinarians and ranchers in endemic areas should keep anthrax in mind as they interact with ill members of susceptible species. Doing otherwise might result in inadvertent exposure to anthrax. A survey of ranchers in the outbreak area is planned by TAHC to assess knowledge, attitudes, and practices regarding anthrax, including information on livestock vaccination.

Recent federal anthrax guidance has focused on the treatment of systemic anthrax, including meningitis, rather than on the more common cutaneous form of the disease. Given that half the cases in the 2001 anthrax incident in the United States (*37*) were cutaneous anthrax and most sporadic cases in the United States and worldwide are cutaneous, this article provides an overview of prevention and control measures for animals and a single resource for the prevention, diagnosis, infection control, and treatment of naturally acquired cutaneous anthrax.
